# Mammogram mastery: A robust dataset for breast cancer detection and medical education

**DOI:** 10.1016/j.dib.2024.110633

**Published:** 2024-06-17

**Authors:** Karzan Barzan Aqdar, Rawand Kawa Mustafa, Zhiyar Hamid Abdulqadir, Peshraw Ahmed Abdalla, Abdalbasit Mohamad Qadir, Alla Abdulqader Shali, Nariman Muhamad Aziz

**Affiliations:** a Department of Computer Science, College of Science and Technology, University of Human Development, Sulaimaniyah, Iraq; b Computer Science Department, College of Science, University of Halabja, Kurdistan Region, Halabja, Iraq; c Consultant Radiologist, X-ray Department, Sulaimani Polytechnic University, Kurdistan Region, Sulaimaniyah, Iraq; d IRO, University of Halabja, Kurdistan Region, Halabja, Iraq; e Consultant Breast Surgeon and Specialist, Faruk Medical City, Sulaimaniyah, Iraq

**Keywords:** Breast cancer, Artificial intelligence, Machine learning, Mammography, Deep learning, Image augmentation

## Abstract

This data article presents a comprehensive dataset comprising breast cancer images collected from patients, encompassing two distinct sets: one from individuals diagnosed with breast cancer and another from those without the condition. Expert physicians carefully select, verify, and categorize the dataset to guarantee its quality and dependability for use in research and teaching. The dataset, which originates from Sulaymaniyah, Iraq, provides a distinctive viewpoint on the frequency and features of breast cancer in the area. This dataset offers a wealth of information for developing and testing deep learning algorithms for identifying breast cancer, with 745 original images and 9,685 augmented images. The addition of augmented X-rays to the dataset increases its adaptability for algorithm development and instructional projects. This dataset holds immense potential for advancing medical research, aiding in the development of innovative diagnostic tools, and fostering educational opportunities for medical students interested in breast cancer detection and diagnosis.

Specifications Table

**Table:** 

Subject	Computer Vision and Pattern Recognition
Specific subject area	Machine Learning; Breast Cancer Detection; Image Processing
Data format	Raw Cleaned Annotated Augmented
Type of data	Image (.JPG files)
Data collection	The dataset consists of 745 original breast X-ray images that have been validated by radiologists and specific doctors. The images were obtained in four mammography units in Sulaimani city, Kurdistan region, Iraq. Images were obtained using a breast X-ray (featured special accessories to limit X-ray exposure to only the breast). All the images are in JPG format and 1920 × 1080 pixels.
Data source location	Institution: Faruk Medical City City/Town/Region: Sulaymaniyah/Malik Mahmood Circle Street Country: Iraq Institution: Breast Disease Treatment Centre City/Town/Region: Sulaymaniyah/Malik Mahmood Circle Street Country: Iraq Institution: Dr. Kalthum Abdula Sofi Clinic City/Town/Region: Sulaymaniyah/Ibrahim Pasha Street Country: Iraq Institution: Dr. Alla Abdulqader Shali Clinic City/Town/Region: Sulaymaniyah/Goran Street Country: Iraq
Data accessibility	Mammogram Mastery: A Robust Dataset for Breast Cancer Detection and Medical Education [1]. Repository name: Mendeley Data Data identification number: DOI: 10.17632/fvjhtskg93.1 Direct URL to data: https://data.mendeley.com/datasets/fvjhtskg93/1

## 1 Value of the Data

• Images in the dataset can be used in image processing and artificial intelligence (AI) research. Furthermore, in the area of breast cancer detection and diagnosis, these data provide high-quality mammography images with two main classes for cancer and non-cancer.
• Although medical professionals and researchers will benefit from these images for detecting breast cancer via machine and deep learning approaches, the dataset can also be used to educate and train medical students. Other researchers can leverage this dataset to develop and validate their own predictive models, contributing to advancements in automated diagnostic tools.
• The dataset is expanded by incorporating data augmentation, increasing the quantity of images. This enables the models to acquire additional pictures depicting diverse situations and perspectives, enhancing their ability to classify unseen images accurately.
• Researchers can reuse this dataset or combine it with other datasets in the field of machine learning to build more accurate models for breast cancer detection since all the images are approved and clear.

## 2 Background

This dataset of mammography images was assembled with the goal of improving early identification and diagnosis of breast cancer, which is the primary cause of death for women. Recently, AI software has received interest as a potentially helpful tool to assist radiologists in decreasing workload and improving diagnostic accuracy. The importance of utilizing machine learning algorithms for accurate diagnosis based on mammography images is highlighted by theoretical and methodological considerations. The dataset is curated to support the development of robust models capable of identifying subtle patterns indicative of breast cancer, thereby aiding medical professionals in timely and accurate diagnoses. The representativeness and usefulness of the dataset for training and validating machine learning algorithms are enhanced by the inclusion of a variety of cases and imaging variations. This dataset article is a useful tool for researchers as it offers standardized mammography data for benchmarking and advancing the field of machine learning-based breast cancer detection.

## 3 Data Description

Globally, breast cancer is the second leading cause of mortality for women. An early and accurate diagnosis of breast cancer is necessary for appropriate treatment planning to save a life [2]. Significant improvements in morbidity and mortality have resulted from annual mammography screening for breast cancer, with a decrease in the incidence of advanced breast cancer of 25.0 percent and a reduction in death of 41.0 percent [3]. The majority of cases of breast cancer occur in women. The condition is a tumor or lump formed by the irregular and uncontrollable growth of breast tissues [4].

Mammography is a diagnostic procedure that uses X-ray technology to distinguish between the rates at which fibro-glandular tissue (FGT) and fatty tissue within the breast are absorbed. Since the breast comprises soft tissues, it is usually exposed to low-energy radiation, typically between 25 and 35 kV. Mammography was previously as simple as pressing an X-ray tube up against the breast and placing a film on the other side. Currently, the process is performed using specialized mammography equipment. These contemporary devices include a compression mechanism in addition to the detector and X-ray tube. This process helps to improve the quality of the generated images by flattening the breast against the detector plate, which encourages a more uniform distribution of FGT. Furthermore, compression lowers the dose needed to obtain high-quality pictures, which are mostly based on breast thickness. Motion artifacts that could normally obfuscate image information are also reduced by the compression [5].

Breast imaging of women between the ages of 34 and 88 was included in the dataset, which was gathered at four separate hospitals and clinics: Faruk Medical City, Breast Disease Treatment Centre, Dr. Kalthum Abdula Sofi Clinic, and Dr. Alla Abdulqader Shali Clinic.

**Fig. 1:**
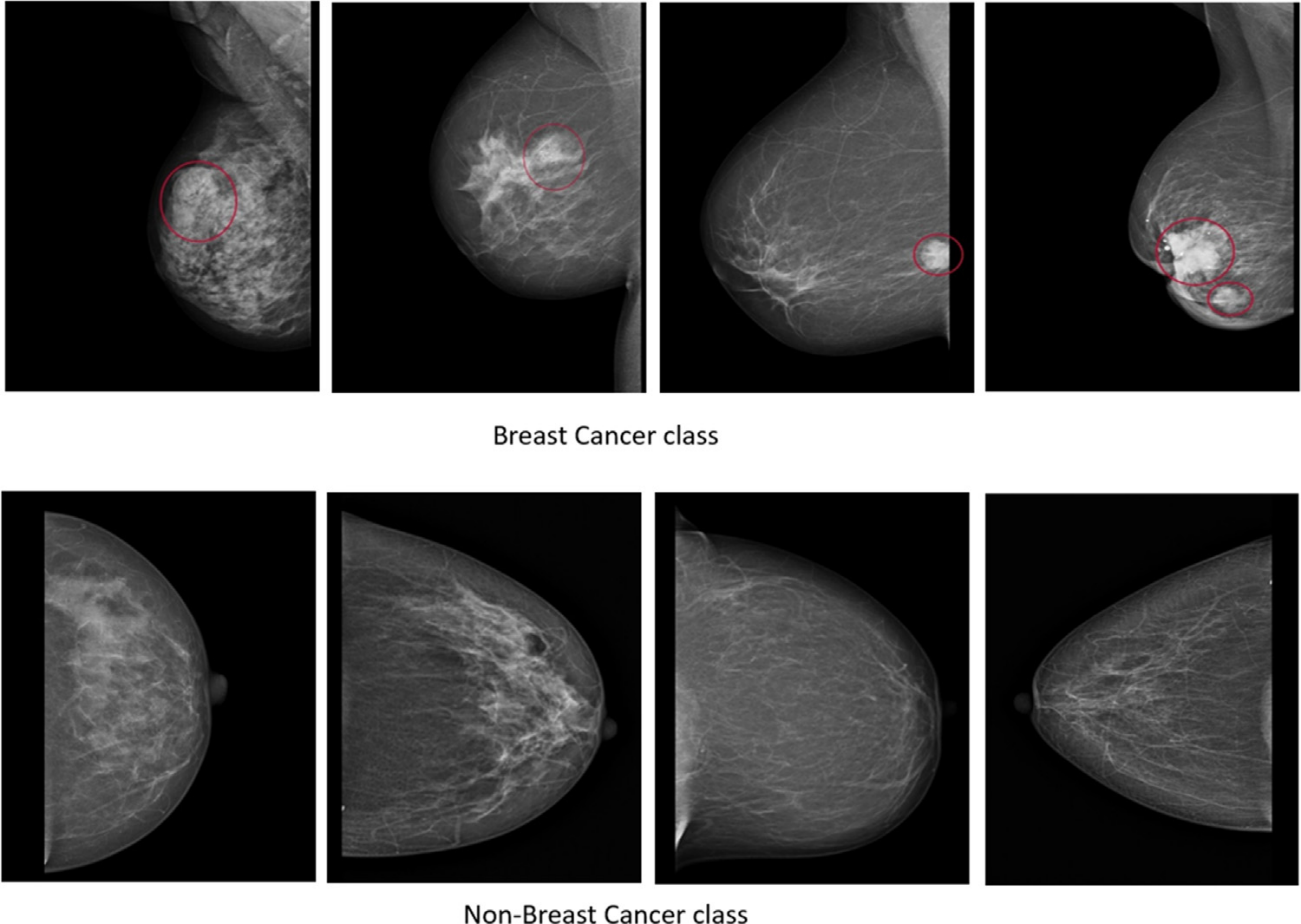
Samples from the original dataset.

The images were gathered during the period spanning 2023–2024. Each image in the dataset is related to a specific person, meaning that a total of 745 female patients were included. The original dataset comprises 745 images (125 cancer and 620 non-cancer), each with an average size of 1920 × 1080 pixels and a dpi ranging from 72 to 96 with a 24-bit depth. All images are saved in JPG format and are classified into two categories: cancer and non-cancer. The distribution of images in each class is illustrated in Table 1, while Fig. 1 provides a visual depiction of the original data samples. Arranged in a 2 × 4 grid format, the first row features original images from the cancer category, while the second row exhibits original images from the non-cancer category. These original images serve as the foundation for subsequent augmentation techniques, enabling the generation of diverse data samples to enhance dataset variability and model generalization. The dataset was carefully collected, annotated, and validated by specialist radiologists and doctors.

**Table 1:** The source and distribution of images, along with the corresponding quantity for each case.

No.	Place	Name of the machine	Quantity of data		Type
1	Faruk Medical City	Siemens MAMMOMAT Inspiration	72		Cancer
2	Breast Disease Treatment Centre	GE Senographe DS Digital Mammography	30		Cancer
3	Dr. Kalthum Abdula Sofi Clinic	Philips MammoDiagnost DR Mammography	562	23	Cancer
				539	Non-Cancer
4	Dr. Alla Abdulqader Clinic	Siemens MAMMOMAT Inspiration	81		Non-Cancer
			745

## 4 Experimental Design, Materials, and Methods

### 4.1 Data collection

At the outset of the first data collection stage, formal consent was sought and obtained from relevant hospitals and medical centers to initiate the data collection process. Subsequently, mammography images were acquired utilizing dedicated equipment within the healthcare facilities. These images, capturing detailed representations of breast tissue, served as the primary source of data for subsequent analysis. The primary dataset consists of 745 images, distributed as 125 cancer and 620 non-cancer instances. Each image has an average dimension of 1920 × 1080 pixels and a dpi varying from 72 to 96 with a 24-bit depth. The mammographs are gathered via four different centers, as detailed in Table 1.

Following image acquisition, the data underwent rigorous examination and analysis by qualified medical professionals, including doctors and specialists trained in the diagnosis of breast conditions. Leveraging their expertise and diagnostic capabilities, these professionals meticulously reviewed and interpreted the mammography images to distinguish between cases indicative of breast cancer and those presenting as non-cancerous conditions.

This multi-step process, involving meticulous consent procedures, image acquisition, and expert analysis, ensured the integrity and reliability of the data collected for the study, laying the groundwork for comprehensive research and analysis in the field of breast cancer detection and diagnosis.

### 4.2 Image augmentations

To augment the dataset, various image augmentation techniques, such as rotation, flipping, zooming, and shifting, can be applied to build deep learning models for the processing of medical pictures. Augmentation approaches are essential and play an important role in both broadening the training dataset and enhancing the model's ability to generalize and adjust to differences found in real-world clinical settings [6]. For this purpose, we provided a Python script using the ***imgaug*** library, which is a powerful library for image augmentation. Twelve augmentation techniques are applied sequentially, with each technique introducing specific variations and distortions to the images, ultimately enhancing the diversity of the dataset and improving the robustness of machine learning models trained on it. The applied augmentation techniques are explained below:

1. **Fliplr**: This technique horizontally flips the images with a probability of 1 (100 % probability), effectively mirroring them along the vertical axis.
2. **Rotate**: Randomly rotate the images within the range of −45 to 45°. This introduces variations in the orientation of the images.
3. **GaussianBlur**: Applies Gaussian blur to the images with a random sigma value within the range of 0–2.0. Gaussian blur smoothens the images, reducing noise and enhancing edges.
4. **AdditiveGaussianNoise**: Adds Gaussian noise to the images with a random scale within the range of 0–0.2 times 255. Gaussian noise introduces random variations in pixel values, simulating noise commonly found in images.
5. **Dropout**: Randomly sets a fraction of pixels in the images to zero with a probability within the range of 0–0.2. Dropout simulates missing or corrupted data, encouraging the model to be more robust.
6. **Resize**: Randomly scales the images to heights and widths within the range of 0.5–1.5 times their original size. This alters the aspect ratio and scale of the images.
7. **Crop**: Randomly crops a portion of the images with a percentage within the range of 0–0.2. Cropping removes parts of the image, focusing on specific regions of interest.
8. **ElasticTransformation**: Applies elastic transformations to the images with a random alpha value within the range of 0–10.0. Elastic transformations distort the images non-rigidly, simulating deformations.
9. **PiecewiseAffine**: Applies piecewise affine transformations to the images with a random scale within the range of 0.02–0.1. Piecewise affine transformations distort the images locally, introducing irregular warping.
10. **PerspectiveTransform**: Applies perspective transformations to the images with a random scale within the range of 0.05–0.15. Perspective transformations simulate changes in viewpoint, skewing the images.
11. **LinearContrast**: Adjusts the contrast of the images linearly with a random factor within the range of 0.2–3.0. Contrast normalization enhances or reduces the difference in intensity between the pixels.
12. **Multiply**: Randomly adjusts the brightness of the images by multiplying the pixel values with a random factor within the range of 0.5–1.5. Multiplying the pixel values changes the overall brightness of the images.

**Fig. 2:**
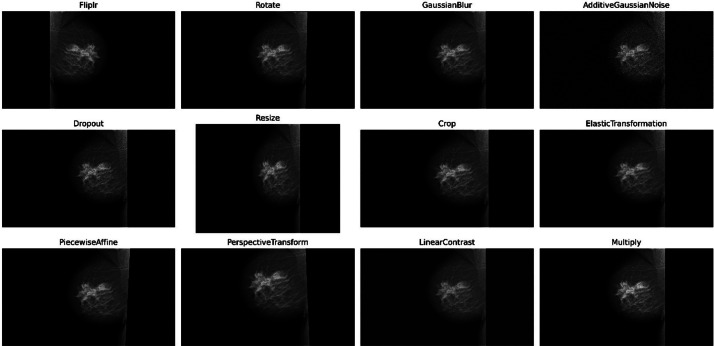
Visual representation of augmented images generated using various techniques.

After applying the augmentation techniques to the original dataset, the number of mammographs has increased significantly. Each original image is augmented to produce 12 additional versions, resulting in a total of 13 images per original sample. Consequently, the number of augmented cancer images has expanded to 1625 and that of non-cancer images to 8060, reflecting the augmented dataset's enriched diversity and expanded size by 9685 images. Table 2 illustrates the changes in the number of X-ray images before and after applying the image augmentation techniques. This augmentation process contributes to enhancing the robustness and generalization capabilities of machine learning models trained on the dataset, ultimately improving their performance in various applications, including breast cancer detection and diagnosis. Samples of images after pre-processing are shown in Fig. 2, the showcased images exhibit a wide range of augmented versions derived from the original dataset using various preprocessing techniques. Each image is clearly labeled with the specific augmentation technique applied, such as horizontal flipping (Fliplr), random rotation (−45 to 45°) (Rotate), Gaussian blur (GaussianBlur), additive Gaussian noise (AdditiveGaussianNoise), pixel dropout (Dropout), random resizing (Resize), random cropping (Crop), elastic transformations (ElasticTransformation), piecewise affine transformations (PiecewiseAffine), perspective transformations (PerspectiveTransform), linear contrast adjustments (LinearContrast), and brightness multiplication (Multiply). These augmentation methods significantly enhance the diversity of the dataset, thereby bolstering its resilience and elevating model performance.

**Table 2:** The changes in the number of X-ray images in the dataset.

Type	Before augmentation	After augmentation
Cancer	125	1625
Non-cancer	620	8060
total	745	9685

### 4.3 Dataset organization

The dataset is organized within a hierarchical structure, comprising a main folder named “breast cancer dataset.” This main folder encompasses two subordinate folders: “original dataset” and “augmented dataset.”

The ``original'' folder is further divided into two subfolders: ``cancer'' and ``non-cancer,'' each housing mammograms without any image processing. The ``cancer'' subfolder contains 125 images, while the ``non-cancer'' subfolder comprises 620 images, resulting in a total of 745 original images within the dataset.

Concurrently, another folder named ``augmented'' resides alongside the main folder. Within the ``augmented'' folder, two subfolders exist: ``cancer'' and ``non-cancer.'' Unlike the original images, the images within the ``augmented'' folder undergo augmentation processes, resulting in an increase in the total number of images.

The augmentation process expands the dataset to a total of 9685 images, with 1625 images representing cancer cases and 8060 images representing non-cancer cases.

**Fig. 3:**
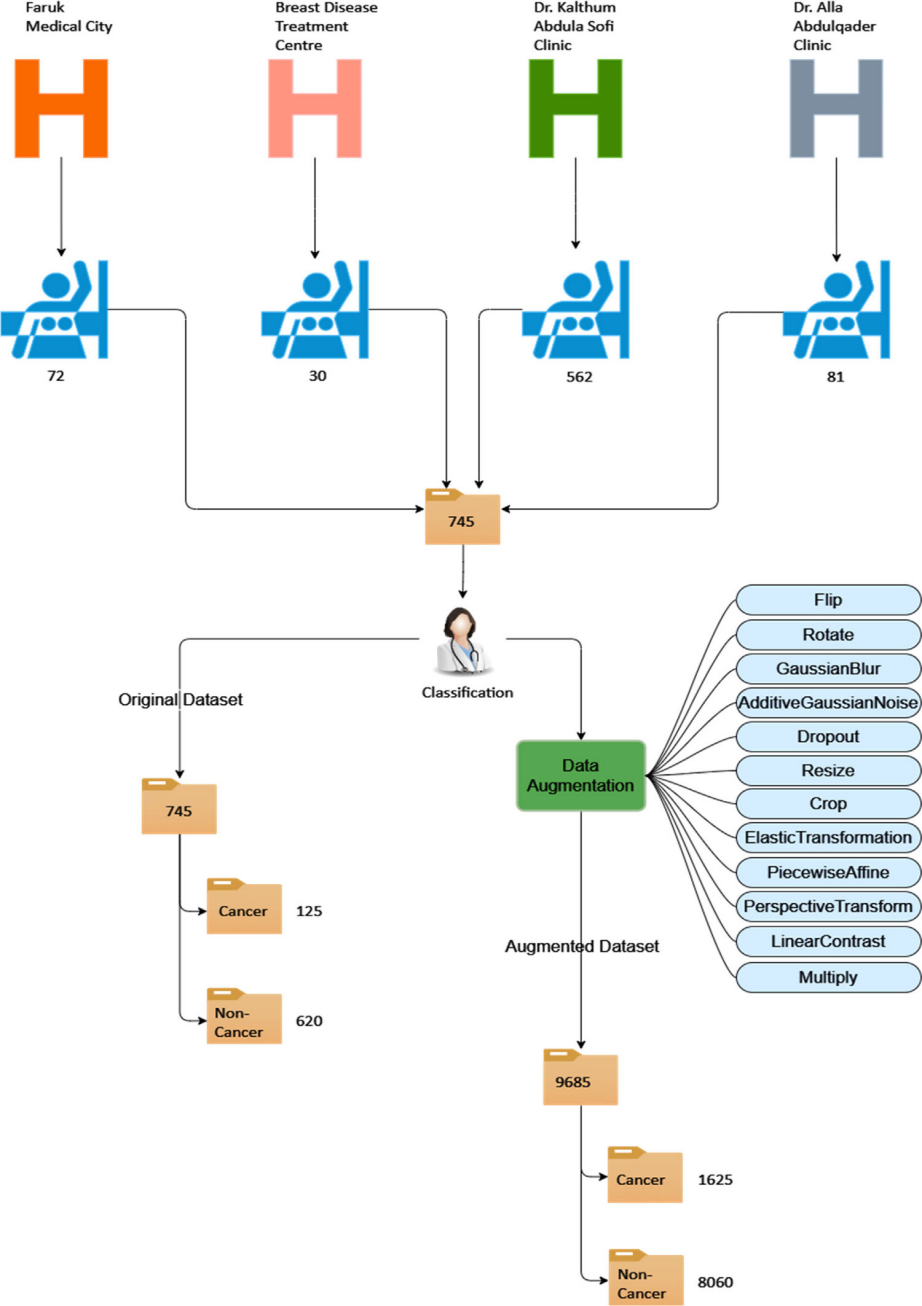
Visual abstract.

Fig. 3 elucidates the comprehensive methodology employed in crafting the dataset, encompassing various stages, such as data collection, image classification, validation, and data augmentation. Additionally, it delineates the structured organization of the entire dataset, delineating the relationships between the main folder, original and augmented subfolders, and their respective contents.

As depicted in Fig. 3, the original mammograms originate from four distinct sources. Specifically, 745 images were collected from these sources, comprising 72 images from Faruk Medical City, 30 images from the Breast Disease Treatment Centre, 562 images from Dr. Kalthum Abdula Sofi Clinic, and 81 images from Dr. Alla Abdulqader Clinic. Subsequently, these images were consolidated into a folder designated as the original dataset. Following this aggregation, medical professionals and experts classified the images into two distinct categories, resulting in the division of the dataset into two subfolders: cancer, containing 125 images, and non-cancer, containing 620 images. Thereafter, the original dataset underwent various preprocessing techniques and mechanisms aimed at augmenting both the quantity and quality of the images. Each original image was subjected to 12 image augmentation techniques. Upon completion of these augmentation procedures, a new folder named the augmented dataset was generated, comprising a total of 9685 images. This augmented dataset was further divided into two subfolders: cancer, containing 1625 images, and non-cancer, containing 8060 images.

## Limitations

A significant challenge encountered during our study was the limited technological infrastructure in our city. With minimal utilization of technology and a lack of available data centers, the process of collecting and managing data was prolonged. Additionally, obtaining approval from surgeons, doctors, and radiologists for data acquisition added to the delay. Due to the absence of data centers, we were only able to collect a limited amount of data. However, it is worth noting that with access to robust data centers, the capacity for data collection could have been significantly increased.

## Ethics Statement

Researchers are mindful of the fact that patients have a right to be protected from public scrutiny of their private lives and illnesses. To this end, the researchers ensured that the patients and the hospital were adequately informed about the objective of this study. In addition, every patient's data remains unknown, and their illness status is protected with the utmost confidentiality.

The relevant informed consent was obtained from all parties involved in the collection and use of data for this study. Additionally, patients provided informed consent for the use of their medical images for research purposes. Patient privacy and confidentiality were strictly maintained throughout the study, and any identifiable information pertaining to the patients involved in this study has been anonymized to protect their privacy.

In this study, ethical approval was not required as the article did not involve human participants, animal experiments, or the collection of data from social media platforms. The use of the dataset adhered to ethical guidelines and regulations concerning data handling and research practices. The authors followed institutional policies throughout the study to ensure the ethical conduct of the research.

## CRediT Author Statement

**Peshraw Ahmed Abdalla**: Writing, Reviewing and Editing, Supervision, Methodology, Software; **Karzan Barzan**: Writing Original Draft, Data Curation; **Rawand Kawa**: Data Curation; **Zhiyar Hamid**: Data Curation; **Abdalbasit Mohammed**: Project Administration, Supervision; Software, Writing- Reviewing, and Editing; **Alla Abdulqader**: Consultant Radiologist, Diagnostic, Resources; **Nariman Muhamad**: Validation, Resources.

## Data Availability

Mammogram Mastery: A Robust Dataset for Breast Cancer Detection and Medical Education (Original data) (Mendeley Data).
